# The Dromiusina Bonelli, 1810 of southwestern Saudi Arabia with description of a new species (Coleoptera, Carabidae, Lebiini)

**DOI:** 10.3897/zookeys.771.24165

**Published:** 2018-07-05

**Authors:** Iftekhar Rasool, Mahmoud S. Abdel-Dayem, Ron F.F.L. Felix, Hathal M. Aldhafer

**Affiliations:** 1 King Saud University Museum of Arthropods (KSMA), Plant Protection Department, College of Food and Agriculture Sciences, King Saud University, P.O. Box 2460 Riyadh 11451, Saudi Arabia; 2 Entomology Department, Faculty of Science, Cairo University, Giza 12613, Egypt; 3 Naturalis Biodiversity Center Leiden, The Netherlands

**Keywords:** *Dromius saudiarabicus* sp. n., Dromiusina, revision, Saudi Arabia, taxonomy

## Abstract

In this paper, species of the subtribe Dromiusina Bonelli, 1810 from southwestern Saudi Arabia are revised. Eleven species in six genera (*Calodromius*, *Dromius*, *Mesolestes*, *Metadromius*, *Microlestes*, and *Zolotarevskyella*) are recognized. *Dromius saudiarabicus* Rasool, Abdel-Dayem and Felix, **sp. n.** is newly described species from Rayda Nature Reserve Asir province. The presence of *Metadromius ephippiatus* in Saudi Arabia is doubtful. A key is also provided to genera and species level for Dromiusina of Saudi Arabia.

## Introduction

The Dromiusina Bonelli, 1810 is the third largest subtribe in the tribe Lebiini of the subfamily Lebiinae, encompassing approximately 735 described species (Lorenz 2005). Its members can be recognized by the fused mentum and prementum. Other important characters of these ground beetles are epipleurae incomplete, not passing the apical angles of elytra; last labial palpomeres pointed; claws smooth or sparsely dentate; base of pronotum weakly incised or straight (Ball and Hilchie 1983, Basilewsky 1984).

Dromiusina are distributed worldwide and are currently classified in 48 genera (Lorenz 2005). Jeannel (1949) placed this subtribe in the tribe Dromiines under subfamily Dromiitae (Lebiidae) and omitted *Apristus* Chaudoir, 1846. Habu (1967) changed Jeannel’s classification and ranked Dromiines as a subtribe of the Lebiini, as did Ball and Hilchie (1983). Kabak (2003) enlisted *Apristus* Chaudoir, 1846, *Eremolestes* Maindron, 1905, *Syntomus* Hope, 1838, and *Tilius* Chaudoir, 1876 under Lionychina Jeannel, 1948 in the Palearctic realm. In the recent Catalogue of Palaearctic Coleoptera, Kabak (2017) followed the classification of Bousquet (2012) and listed genera of Lionychina and Singilina under Dromiusina. Macrosystematic issues are very complex and need further investigation at molecular level.

The Dromiusina fauna of the Arabian Peninsula is not completely studied and only 18 species are reported. Five species were listed from United Arab Emirates (Felix 2009), 18 from Saudi Arabia (Britton 1948, Mateu 1979, 1986, 1990, Kabak 2003, Felix 2009), and six from Yemen (Kabak 2003, Felix 2009). Not a single species of Dromiusina has been reported from Kuwait, Oman, or Qatar until now. This subtribe has also been poorly studied in Saudi. The first list of species was given within the Carabiade of southwest Arabia by Britton (1948) who included three species: *Microlests vittatus* Mostschulsky, 1859, *M. micromys* Alluaud, 1918, and *M. discoidalis* Fairmaire, 1892 from Hejaz (Saudi Arabia). Mateu (1979) presented the first synopsis of subfamily Lebiinae that included a list of eight Dromiusina species of Saudi Arabia and the description of one species *Metadromius arabicus*. Mateu (1986) published a list of Lebiinae and Brachininae of Saudi Arabia with four additional species. Mateu (1990) described *Dromius buettikeri* as a new species from Makkah, Harithi, which is thought to be endemic to Saudi Arabia.

The present study is the third in a series of papers revising the southwestern Saudi Arabian Lebiini (Rasool et al. 2017, 2018). A total of eleven species is recognized and treated in subtribe Dromiusina, including one new species. The study includes a key to species of Dromiusina in Saudi Arabia and illustrations of the most important characters.

## Materials and methods

This review is based on extensive surveys (during 2012–2016) in southwestern Saudi Arabia (Al Baha, Asir, and Jizan provinces) and preserved collections at King Saud University Museum of Arthropods, Saudi Arabia (KSMA) (comprising 2253 specimens). Additional materials, including holotypes and paratypes, were also borrowed for examination from the following museums: British Museum of Natural History, London, UK (**BMNH**), Natural History Museum Basel, Switzerland (**NHMB**) and Naturalis Biodiversity Centre, Leiden Netherlands (**RMNH**). The newly collected materials were deposited in KSMA. Other acronyms of the holotype depositories mentioned in the text are the National Museum of Natural History, Paris, France (**MNHN**) and the Museum of Natural History, Hungary (**MNH**).

For collection of species, light trap (LT), hand Picking (HP), pit fall trap (PT) and sticky trap (ST) were used. Male specimens of freshly collected species were dissected for aedeagus, which is boiled in 70 % KOH for 1–2 minutes to eliminate additional tissues and kept in clove oil for 24 hours. The aedeagus were glued on cards or preserved inside a glycerin vial pinned under specimen.

All the species and aedeagus were photographed by Q–imaging Micro Publisher 5.0 RTV camera, attached with a trinocular stereomicroscope (LEICA MZ125). Taken images were joined by software Zerene Staker 1.04. FEI Inspect S50 model (Scanning Electron Microscope) was used to take scanned images.

Total body length (**TBL**) was measured from the anterior margin of labrum to terminating margin of abdomen along midline; head length (**HL**) was taken from anterior margin of labrum to anterior margin of pronotum along middle line, while pronotum length (**PL**) and elytra length (**EL**) was taken from anterior to posterior margin along the middle line of pronotum and elytra respectively; head width including eyes (**HW**), pronotum width (**PW**) and elytra width (**EW**) were measured at their widest points. Aedeagus length (AL) was measured along its body mass. All the measurements were taken with an ocular micrometer in a stereo-binocular microscope (МБС-9).

Verbatim label data cited for the type specimens of the newly described species have label breaks indicated by a slash (“/”). The chorotypes of species were designated by following the classification of Taglianti et al. (1999). For synonymy and species distribution, Kabak (2003, 2017), Bousquet (2012), Anichtchenko (2017) (http://carabidae.org), and available literature are followed.

## Systematics

### Key to genera and species of Dromiusina of southwestern Saudi Arabia

**Table:** 

1	Antennomeres II shorter than III (Fig. 1)	**2**
–	Antennomeres II as long as III (Figs 2, 3, 4)	**7**
2	Pubescence starts from antennomeres III (Fig. 1); elytra parallel sided; tarsomeres I of hind legs longer than last (Fig. 5)	***Microlestes*, 3**
–	Pubescence starts from antennomeres IV (Fig. 3); elytra parallel sided or broadened posteriorly; tarsomeres I of hind legs as long as last (Fig. 6)	**5**
3	Antennae short and stout, crossing the base of pronotum with two antennomeres; eyes small, tempora long, head with microlines (Fig. 18)	***Microlestes infuscatus fragilis***
–	Antennae long and slender, crossing the base of pronotum by three and half antennomeres; eyes large,tempora short, head without microlines (Figs 16, 17)	**4**
4	Whole of the body black (Fig. 30); elytra with strong transverse microlines; aedeagus in lateral view straight in middle from ventral side, endophallus armature of aedeagus broad and flattened (Fig. 43)	***Microlestes glabrellus***
–	Elytra dark brown, with large elongate and pale testaceous discal spots (Fig. 29), abdomen–dark brown, elytra with suppressed transverse microlines, aedeagus in lateral view strongly curved throughout, endophallus armature of aedeagus elongate (Fig. 42)	***Microlestes discoidalis***
5	Base of pronotum weakly incised towards hind angles (Fig. 11); elytra broadened posteriorly; apex of elytra transversely truncated; elytra pale testaceous with transverse black band in the middle, not reaching to the lateral margins (Fig. 22)	***Calodromius mayeti***
–	Base of pronotum straight (Figs 12, 13); elytra parallel sided, apex of elytra slightly obliquely truncates, elytra usually with pale macula (Figs 23, 24)	***Dromius*, 6**
6	Head wider than long; pronotum strongly transverse, sides of pronotum almost straight posteriorly, hind angles almost right; labrum transverse, tempora short, frons with few transverse wrinkles (Fig. 13)	***Dromius buettikeri***
–	Head longer than wide; pronotum not strongly transverse, narrowed and sinuate posteriorly before angles, hind angles obtusangular; labrum as long as wide, tempora long, frons smooth (Fig. 12)	***Dromius saudiarabicus* sp. n.**
7	Head and pronotum black with longitudinal furrows (Fig. 21); elytra parallel sided, apex transversely truncated; whole of the body glossy (Fig. 36)	***Zolotarevskyella rhytidera***
–	Head and pronotum with microsculptures (Figs 14, 15); elytra broadened posteriorly (Figs 25–28, 33–35), apex of elytra obliquely or transversely truncated	**8**
8	Mentum with medium tooth (Fig. 10); pubescence of antennae starts from antennomeres IV (Fig. 3); apex of elytra obliquely truncate; base of pronotum lobate in the middle (Fig. 14, 15, 28)	***Metadromius*, 9**
–	Mentum without median tooth (Fig. 9); pubescence of antennae starts from antennomeres II (Fig. 4); apex of elytra transversely truncate; base of pronotum straight in the middle	***Pseudomesolestes*, 11**
9	Male with 4 setae at apical margin of last abdominal sternum; head and pronotum densely and coarsely punctate with isodiametric mesh pattern (Figs 7, 15); apical lamina of aedeagus broadly ended (Fig. 41)	***Metadromius brittoni***
–	Male with 2 setae at apical margin of last sternum; head and pronotum finely and sparsely punctate, pronotum and elytra without isodiametric mesh pattern (Figs 8, 14); apical lamina of aedeagus elongate and narrowed (Figs 40)	**10**
10	Pronotum testaceous; head without microsculptures; elytra with sparse pubescence, disc of elytra with transverse dark brown pattern (Figs 25, 26)	***Metadromius arabicus***
–	Pronotum dark brown; head with microsculptures; elytra with dense and short pubescence, disc of elytra with round dark brown pattern (Fig. 28)	***Metadromius* spec.**
11	Head, pronotum and elytra with granulated microsculptures; pronotum with few wrinkles along the medial impression (Fig. 19); femora, maxillary and labial palpi dark brown; apical lamina of aedeagus short. (Fig. 45)	***Pseudomesolestes brittoni***
–	Head, pronotum and elytra with isodiametric mesh pattern; pronotum without wrinkles along the medial impression (Fig. 20); femora, maxillary and labial palpi pale testaceous; apical lamina of aedeagus elongate (Fig. 46)	***Pseudomesolestes quadriguttatus***


**Figures 1–10.**
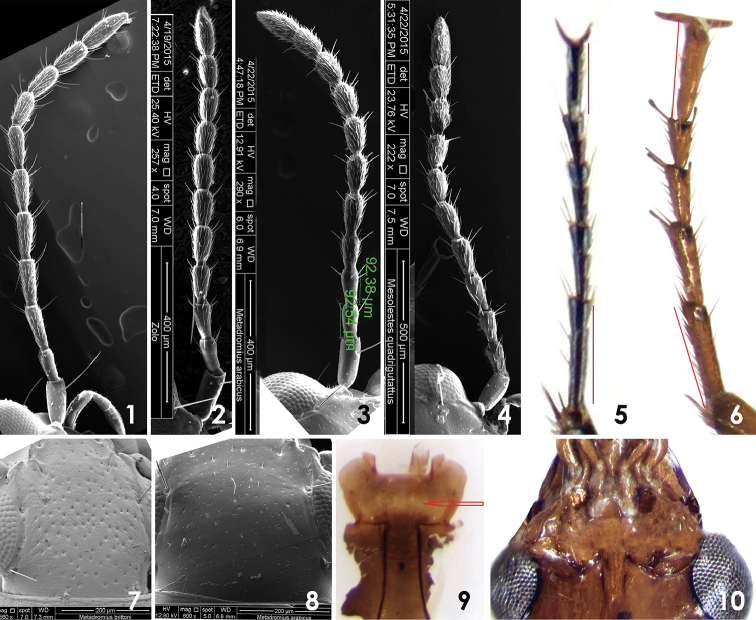
**Different characters used in key: 1, 5** Antennae and legs of *Microlestes discoidalis* (Fairmaire, 1892) **2** Antennae of *Zolotarevskyella rhytidera* (Chaudoir, 1876) **3, 8, 10** Antennae, dorsal view of head and mentum of *Metadromius arabicus* Mateu, 1979 **4, 9** Antennae and mentum of *Pseudomesolestes quadriguttatus* Mateu, 1979 **6** Leg of *Dromius saudiarabicus* sp. n. **7** Dorsal view of head of *Metadromius brittoni* (Basilewsky, 1948).

#### Calodromius

Taxon classificationAnimaliaColeopteraCarabidae

Reitter, 1905

##### Type species.

*Carabus quadrinotatus* Panzer, 1799 (= *Carabus spilotus* Illiger, 1798).

*Calodromius* is poor in species among the subtribe Dromiusina, with only eight species in the world (Anichtchenko 2017), seven species of which are from the Palaearctic realm (Kabak 2003). The genus *Calodromius* can be identified among its related genera by combination of characters: antennomeres II shorter than III; pubescence starts from antennomeres IV; base of pronotum weakly incised towards hind angles; elytra broadened posteriorly; tarsomeres I of hind legs as long as last. *Calodromius mayeti* (Bedel 1907) is the only representative of the genus from Arabian Peninsula, recorded from Madina, Saudi Arabia (Mateu 1986).

#### Calodromius mayeti

Taxon classificationAnimaliaColeopteraCarabidae

(Bedel, 1907)

**Figures 11–21.**
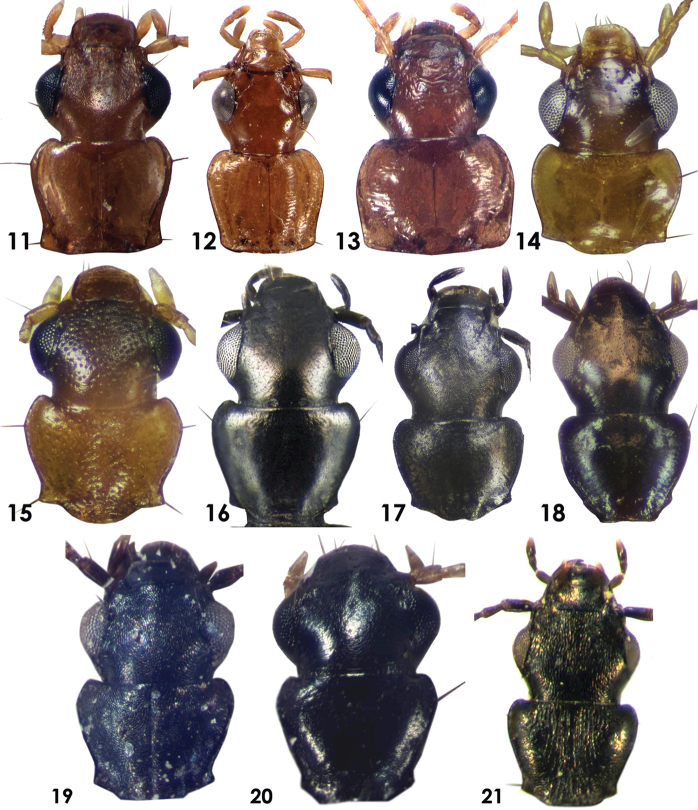
Dorsal view of head and pronotum of Dromiusina species: **11** *Calodromius mayeti* (Bedel, 1907) **12** *Dromius saudiarabicus* sp. n. **13** *D. buettikeri* Mateu, 1990 **14** *Metadromius arabicus* Mateu, 1979 **15** *M. brittoni* (Basilewsky, 1948) **16** *Microlestes discoidalis* (Fairmaire, 1892) **17** *M. glabrellus* (Reitter, 1901) **18** *M. infuscatus fragilis* Mateu, 1956 **19** *Pseudomesolestes brittoni* Mateu, 1956 **20** *P. quadriguttatus* Mateu, 1979 **21** *Zolotarevskyella rhytidera* (Chaudoir, 1876).

**Figures 22–30.**
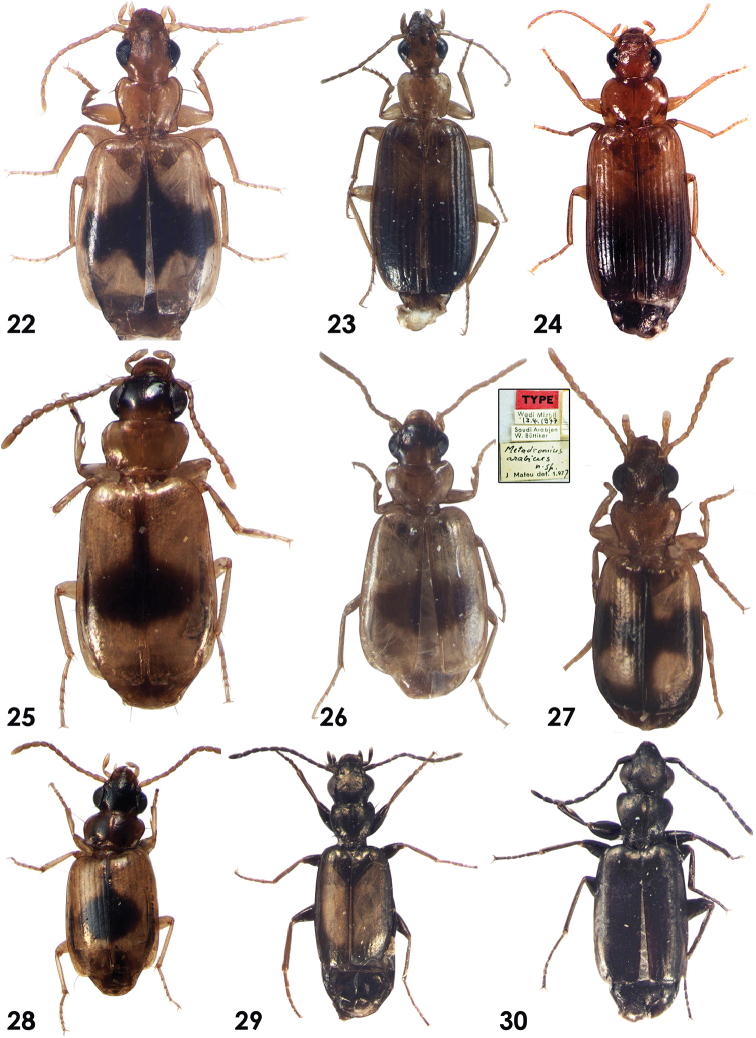
Habitus of Dromiusina species: **22** *Calodromius mayeti* (Bedel, 1907) **23** *Dromius saudiarabicus* sp. n. **24** *Dromius buettikeri* Mateu, 1990 (**25, 26)** *Metadromius arabicus* Mateu, 1979 **27** *Metadromius brittoni* (Basilewsky, 1948) **28** *Metadromius* spec. **29** *Microlestes discoidalis* (Fairmaire, 1892) **30** *Microlestes glabrellus* (Reitter, 1901).

Figures 11 , 22 , 37 , 49

Dromius mayeti Bedel, 1907: 272.

##### Type locality.

Tunisia.

##### Type depository.

Holotype male in MNHN: Paratype in NHMB

##### Material examined.

Total 21 specimens: 1♀ “[yellow label]” / “Saudi Arabia, W. Buttiker” / Butayn, 21.IV.1981”/ “*Philorhizus mayeti*, J. Mateu det. 1983”. [**NHMB**]. Al Baha: 1♀, “KSA, Al Makhwa, Shada Al Aala, 19°52.598'N 41°18.672'E Alt. 892 m, 26.I.2015, (HP on light), I. Rasool”. 2♂, “16.II.2014, (LT), M.S. Abdel-Dayem & I. Rasool”. 1♂, 2♀, “19°51.066'N 41°18.037'E Alt. 1325 m, 02.III.2015, (LT)., 1♀, “19°51.066'N 41°18.037'E Alt. 1325 m, 17.X.2014, (LT)., 1♀, “19°50.710'N 41°18.267'E Alt. 1474 m, (LT), H. Al Dhafer, M.S. Abdel-Dayem, H. H. Fadl & I. Rasool”. 1♂, 2♀, “19°52.717'N 41°18.712'E Alt. 825 m, 15.XI.2015, (LT)., 1♂, 1♀, “13.XI.2015, (LT)., 2♀, “19°52.598'N 41°18.672'E Alt. 892 m, 13.XI.2015, (LT)., 1♂, 1♀, “19°51.762'N 41°18.089'E Alt. 1225, 12.XI.2015, (LT)., 1♀, “19°52.685'N 41°18.663'E Alt. 851 m, 15.XI.2015, (LT)., 1♂, “19°51.066'N 41°18.037'E Alt. 1325 m, 14.XI.2015, (LT), H. Al Dhafer, M.S. Abdel-Dayem, H. H. Fadl,A. Elgarbawy, El Turkey and Soliman, A.” Asir: 1♂, “Asir, Abha, Rayda, 18°12.315'N 42°24.607'E Alt. 2578 m, 18.XI.2015, (LT), H. Al Dhafer, M.S. Abdel-Dayem, H. H. Fadl, A. Elgarbawy, El Turkey and Soliman” [**KSMA].**

##### Description.

Body form (Fig. 22), small species 3.60–3.90 mm. *Color*: Dorsum and ventrum of pronotum and abdomen, epipleurae, antennae, mouthparts and legs testaceous; head slightly darker than others; elytra pale testaceous with transverse black band in the middle, not reaching to the lateral margins, black band prolonged to the base along the suture, shortly extended towards the apex. *Microsculpture*: head, labrum, pronotum and elytra with isodiametric mesh pattern; sternite with transverse microlines. *Head*: almost as long as wide, HL 0.72–0.82 mm and HW 0.70–0.79 mm (Fig. 11). *Pronotum*: transverse, wider than long, PW 0.71–0.78 mm, and PL 0.56–0.64; sinuate posteriorly, base almost straight with acute angles (Fig. 11). *Elytra*: broadened posteriorly, EL 1.87–2.06 mm and EW 1.42–1.49 mm; apical margins transversely truncate; striae and intervals finely punctuate, provided with short brown pubescence. Claws smooth. *Abdomen*: All visible sternite with short pubescence, apical margin of last sternum rounded and 4–setose in both sexes. *Aedeagus*: Small and thick aedeagus (Fig. 37), 0.78 mm; basal side of aedeagus narrowed; very broad and depressed at apical lamina; apical end short and with tooth like tip.

##### Ecological notes.

This species was collected in the natural habitat of mountains and valleys covered with variety of vegetation, sand, and stones. Species was distributed in elevation ranging from 892–1611 m (Fig. 49). Adult beetles were attracted to UV–light. In winters this species appears in low elevation while in summers it appears in high elevation.

##### Geographical distribution.

This species is recorded from Iran, Libya, Morocco, Saudi Arabia, Tunisia, UAE (Kabak 2003, 2017, Felix 2009). In current study it is collected from Al Baha and Asir regions of Saudi Arabia. It is a Mediterranean species that exemplifies Mediterranean-Sindian chorotype.

#### Dromius

Taxon classificationAnimaliaColeopteraCarabidae

Bonelli, 1810

##### Type species.

*Carabus quadrimaculatus* Linné, 1758.

The genus *Dromius* is type genus of subtribe Dromiusina, representing 105 species in the world (Lorenz 2005) distributed in four subgenera (Anichtchenko 2017). These species are distributed in almost all zoogeographical regions, Nearctic, Neotropical, Australian, Oriental, Afrotropical, and Palaearctic regions. In the Palaearctic it is represented by 53 species (Bousquet 2012). This genus can be differentiated from other genera in the subtribe Dromiusina by elongate and parallel sided elytra; head small and with constricted neck; labrum semirounded, mentum without tooth; antennae long and cylindrical, pubescence starts from antennomeres IV; base of pronotum straightly truncate, and pronotum sometimes slightly sinuate posteriorly; tarsomeres I–III dilated in fore legs, basal tarsomere of hind legs as long as last, claws dentate; elytra usually with pale macula, apical margins of elytra obliquely truncate (Lindroth 1974, Bousquet 2010). Only *D. buettikeri* Mateu, 1990 is described from Saudi Arabia and D. (Dromius) saudiarabicus sp. n. is described in the present work from Asir Province.

#### Dromius saudiarabicus

Taxon classificationAnimaliaColeopteraCarabidae

, Rasool, Abdel-Dayem & Felix sp. n.

http://zoobank.org/47D2FBB7-38D4-4199-8A8E-09A2777F0552

Figures 6 , 12 , 23 , 38 , 48 , 50

##### Type material.

23 specimens: HOLOTYPE, male in KSMA, point-mounted, labeled: “KSA, Asir, Abha, Rayda, 18°11.695'N 42°23.818'E Alt. 1897 m, 21.X.2014, (LT), H. Al Dhafer, M.S. Abdel-Dayem, H.H. Fadl, A. El Turkey & A. Elgarbawy” / “Holotype [red label] *Dromius saudiarabicus* sp. n.” [printed label]. Paratypes: Total 22 specimens, sex and label data as follows. “4♀, same as holotype”. 1♀, same as holotype except, 18.XI.2015, H. Al Dhafer, M.S. Abdel-Dayem, H.H. Fadl, A. El Turkey, A. Elgarbawy & Soliman, A”. 1♂, 1♀, same as holotype except, “18°12.315'N 42°24.607'E Alt. 2761 m, 30.II.2014., “1♀, same as holotype except “18°11.766'N 42°24.315'E Alt. 2285 m, 20.X.2014., 2♀, same as holotype except “18°11.884'N 42°24.435'E Alt. 2387 m, 20.X.2014., 1♂, 1♀, same as holotype except “18°12.315'N 42°24.607'E Alt. 2761 m, 20.X.2014., 2♂, same as holotype except “18°12.095'N 42°24.536'E Alt. 2578 m, 20.X.2014., 1♂, 1♀ same as holotype except, “18°12.095'N 42°24.536'E Alt. 2578 m, 18.XI.2015, H. Al Dhafer, M.S. Abdel-Dayem, H.H. Fadl, A. El Turkey, A. Elgarbawy & Soliman, A”. 2♂, 2♀ same as holotype except, “18°11.766'N 42°24.315'E Alt. 2285 m, 18.XI.2015, H. Al Dhafer, M.S. Abdel-Dayem, H.H. Fadl, A. El Turkey, A. Elgarbawy & Soliman, A” [**KSMA**]. 1♀ same as holotype except, “18°12.315'N 42°24.607'E Alt. 2761 m, 20. X.2014., 1♂, same as holotype except, “18°11.884'N 42°24.435'E Alt. 2387 m, 20.X.2014” [**RMNH**]. All paratypes with second label reading “Paratype *Dromius saudiarabicus* sp. n.” [yellow label]

##### Type locality.

Rayda Nature Reserve (18°12'N, 42°24'E), 20 km northwest the city of Abha, Asir Province, southwestern Saudi Arabia.

##### Specific epithet.

The specific epithet is a Latinized adjective in the masculine form based on country Saudi Arabia, from which the new species is described.

##### Diagnosis.

Adults of *Dromius saudiarabicus* sp. n. have all the features of other members of subgenus Dromius Bonelli, 1810 and can be distinguished from them by the following combination of external features: dorsum of head and pronotum rufous to rufo-testaceous, Elytra dark brown, with testaceous maculae, antennae, mandibles, palpi, and legs; head without microlines, but with mesh pattern isodiametric on the vertex, frons smooth; head longer than wide; tempora long with strongly constricted neck; pronotum not strongly transverse, narrowed and sinuate posteriorly before angles.

##### Description.

*Habitus*: Body form (Fig. 21) elongate subparallel sized species, TBL Holotype 6.90 mm, male 6.80–7.30 mm, female 7.00–8.00 mm. *Color*: Dorsum of head and pronotum rufous to rufo–testaceous; antennae, mandibles, palpi and legs testaceous. Elytra dark brown, with testaceous macula suture below scutellum with different expending range, but never reaching middle or lateral border of elytra; epipleurae testaceous anteriorly and dark brown posteriorly; ventrum of thorax testaceous, abdominal sternites dark brown laterally and testaceous medially, sometimes sternites III–V completely dark brown. *Microsculpture*: Head without microlines, but with mesh pattern isodiametric on the vertex, frons smooth; pronotum with distinct transverse wrinkles medially along the median longitudinal impression, smooth laterally; elytra with mesh pattern isodiametric, microlines absent; thoracic ventrum smooth; abdomen with microlines. *Luster*: Head, pronotum and ventrum glossy, elytra moderately dull. *Head*: Small and obtuse (Fig. 12), Holotype HL 1.36 mm and HW 1.16 mm; tempora long with strongly constricted neck; surface smooth with two pairs of supraorbital setae; clypeus smooth, larger than labrum, with a pair of setae; labrum almost as long as wide, rounded laterally, with anterior margins slightly convex; last segments of maxillary and labial palpi pointed; mentum without median tooth; antennae long filiform, extending beyond base of pronotum by three antennomeres; antennomeres I, III and IV equal in length and longer than the rest; antennomeres II shortest; antennomeres V–IX subequal; pubescence starts from antennomere IV. *Pronotum*: more or less transverse, (Fig. 12) Holotype PL 1.05 mm and PW 1.24 mm; median longitudinal impression deep; narrowed posteriorly, slightly sinuate before basal angles; provided with two pairs of lateral setae; anterior margins of pronotum concave with rounded angles and basal margins straight with almost right angles. *Elytra*: subparallel sided, Holotype WL 4.10 mm and EW 2.50 mm, broadened in the posterior third; humeri broadly rounded; striae clear; apices of elytra slightly obliquely truncate; epipleurae ends before apical angles of elytra. *Legs*: Long and slender; protarsomeres I–III dilated in male; tarsomeres I shorter than V in fore legs, tarsomeres I as long as V in median and hind legs; claws with 2–3 tooth in the middle. *Abdomen*: abdominal sternite smooth, laterally pubescent; suture between III and IV sternite not complete; margins of last two sternite with lateral setae, last sternum emarginated medially in males and rounded in females; males and females with 8 anal setiferous punctures at the apical margin of the last sternum, 4 inner setiferous punctures shorter than outer in females. *Aedeagus*: Shape of aedeagus (Fig. 38), AL of Holotype 1.50 mm; in lateral view it is narrowed at both ends, broad in the middle, curved dorsally; apical lamina long, narrowed and depressed, rounded apically; basal side rounded and cylindrical.

##### Affinities.

Externally, *Dromius saudiarabicus* sp. n. is similar to *D. buettikeri* Mateu, 1990 and *D. meridionalis* Dejean, 1825, but it can be differentiated from both species by its dull surface, constricted neck, and shape and internal sac of aedeagus. It can also be separated from *D. buettikeri* by its comparatively less transverse pronotum, sinuate lateral margin of pronotum and tempora long. It is also separated from *D. meridionalis* by its slightly sinuate lateral margin of pronotum and absence of two ridges near eyes.

##### Ecological notes.

This species was collected at elevation of 1897–2761 m (Fig. 50) in Rayda Nature Reserve. Fully winged beetles were collected by UV–light traps from steep slopes covered in woodlands dominated by juniper *Juniperus procera* Hochst. Ex Endl. (Cupressaceae) (Fig. 48) and wild olive trees, *Oleae europaea cuspidata* (Wall. ex G. Don) Cif. (Oleaceae). Adults are collected only during October.

##### Geographical distribution.

This species is only known from the type locality in the Rayda Nature Reserve, Abha, on the southwestern edge of Al Souda Mountain, in the Asir Highlands of the southwestern of Saudi Arabia (Fig. 50).

#### Dromius buettikeri

Taxon classificationAnimaliaColeopteraCarabidae

Mateu, 1990: 40

Figures 13 , 24 , 39 , 50

Dromius buettikeri Mateu, 1990: 40.

##### Type locality.

Saudi Arabia, Makkah, Harithi.

##### Type depository.

Holotype male and paratypes one male and one female in NHMB.

##### Material examined.

Total 22 specimens: Al Baha: 1♀, “KSA, Al Baha, Al Makhwa, Shada Al Aala, 19°50.710'N E41°18.267'E Alt. 1474 m, 27.I.2014, (LT)., 1♀, “19°50.575'N 41°18.691'E Alt. 1666 m, 27.I.2015, (LT)., 1♀, “19°50.411'N 41°18.686'E Alt. 1611 m, 27.I.2015, (LT)., 1♂, “19°50.329'N 41°18.604'E N 41°18.604'E Alt. 1563 m, 27.I.2015, (LT)., 1♀, “19°51.066'N 41°18.037'E Alt. 1325 m, (LT)., 1♂, 1♀, “19°50.710'N 41°18.267'E Alt. 1474 m, 15.II.2014, (LT), H. Al Dhafer, M.S. Abdel-Dayem, H. H. Fadl, A. El Turkey, A. Elgarbaway & I. Rasool”. 1♂, “19°52.717'N 41°18.712'E Alt. 825 m, 15.XI.2015, (LT)., 1♀, “13.XI.2015, (LT)., 1♀, “19°52.685'N 41°18.663'E Alt. 851 m, 15.XI.2015, (LT)., 1♂, 1♀, “19°52.598'N 41°18.672'E Alt. 892 m, 12.XI.2015, (LT)., 1♀, “19°51.066'N 41°18.037'E Alt. 1325 m, 15.XI.2015, (LT)., 1♂, 2♀, “14.XI.2015, (LT)., 1♂, “9°50.710'N 41°18.267'E Alt. 1474 m, 14.XI.2015, (LT), H. Al Dhafer, M.S. Abdel-Dayem, H. H. Fadl, A. El Turkey, A. Elgarbaway & Soliman, A”. 1♂, “19°50.710'N 41°18.267'E Alt. 1474 m, 08.XII.2014, (LT), H. Al Dhafer, M.S. Abdel-Dayem, H.H. Fadl, A. El Turkey, A. Elgarbaway & I. Rasool”. Asir: 1♀, “KSA, Abha, Wadi Rayda, 18°11.749'N 42°23.345'E Alt. 1614 m, 24.II.2014, (LT), I. Rasool.” 1♂, “17.XI.2015, (LT)., 1♀, “18°11.749'N 42°23.345'E Alt. 1614 m, 11.XII.2014, (LT)., 1♂, “18°11.679'N 42°23.691'E Alt. 1851 m, 11.XII.2014, (LT), H. Al Dhafer, M.S. Abdel-Dayem, H.H. Fadl, A. El Turkey, A. Elgarbaway & I. Rasool” [**KSMA].** 1♂, “KSA, Abha, Wadi Rayda, 18°11.749'N 42°23.345'E Alt. 1614 m, 24.II.2014, (LT), I. Rasool” [**RMNH].**

##### Description.

Elongate and parallel sized species (Fig. 24), 5.50–6.30 mm. *Color*: Head, pronotum and basal four sternite light brown in the middle; mouth parts, antennae, legs, anterior 3/4 of epipleurae testaceous; humeri with pale or testaceous macula may extend to middle of elytra; rest of elytra, apical fourth of epipleurae and lateral boarders of elytra, last two abdominal sternite dark brown. *Microsculpture*: Head, pronotum, elytra with isodiametric mesh pattern; abdomen with depressed microlines. *Head*: Wider than long, HL 0.92–1.06 mm, HW 1.05–1.12 mm, narrower than pronotum; tempora short and curved (Fig. 13). *Pronotum*: Broad, transverse, PL 0.99–0.92 mm, PW 1.23–1.34 mm, lateral margins almost straight, basal angles right with straight base (Fig. 13). *Elytra*: Elongate and parallel sized, WL 3.25–3.75 mm, EW 1.87–2.12 mm. Claws with 2–3 teeth in the middle. *Abdomen*: last two sternite with 8 setiferous setae in females and 4 in males; lateral margins with fine scattered pubescence. *Aedeagus*: narrowed at both ends, AL 1.09 mm. In lateral view, it is incised in the middle from ventral sides and hump like from dorsal sides, broad in the middle. Tip of apical lamina short and rounded, weakly incised near dorsal margin of apical lamella (Fig. 39).

##### Ecological notes.

Adult beetles were collected by UV–light from elevation ranges from 1474–1851 m on steep sloop mountains (Fig. 50), characterized by surface vegetation, stones, gravels and small shrubs and trees, this species was collected in December to February in winter season.

##### Geographical distribution.

Endemic to the Saudi Arabia and only found in nature reserve of Rayda mountains in Abha, Asir (Mateu 1990, Kabak 2017).

#### Metadromius

Taxon classificationAnimaliaColeopteraCarabidae

Bedel, 1907

##### Type species.

*Dromius myrmidon* Fairmaire, 1859

*Metadromius* is a complex genus that comprises about 30 species that are distributed in Afrotropical and Palaearctic regions (Anichtchenko 2017). A revision of the genus in the Middle East is in preparation, with some new species. Fifteen species inhabit Palaearctic region, three are mentioned from within the territories of Arabian Peninsula (Mateu 1979, Kabak 2003, 2017 Anichtchenko 2017). The genus can be differentiated from other genera in the subtribe Dromiusina by: small body size range from 2–3 mm; mentum with median tooth; antennae stout, antennomere II as long as III, pubescence starts from antennomere IV; anterior margin of labrum slightly rounded; pronotum transverse, base of pronotum weakly incised, sinuate posteriorly with sharp angles; apex of elytra obliquely truncate; tarsomeres I longer than V in hind legs, claws dentate. In Saudi Arabia, *M. arabicus* Mateu, 1979; *M. ephippiatus* Fairmaire, 1884; and *M. brittoni* Basilewsky, 1948 are mentioned up to now (Mateu 1979, 1986, Kabak 2003). However, the record of *M. ephippiatus* is a doubtful one. Its occurrence is based on a probably false identification by Mateu (1979).

#### Metadromius arabicus

Taxon classificationAnimaliaColeopteraCarabidae

Mateu, 1979

Figures 3 , 8 , 10 , 14

Metadromius arabicus Mateu, 1979: 151.

##### Type locality.

Saudi Arabia, Riyadh, Wadi Mazbil.

##### Type depository.

Holotype male in NHMB.

##### Material examined.

**Holotype.** Total 654 specimens: Male labeled “HOLOTYPE [red label]” / “Saudi Arabien, W. Büttiker” / “Wadi Mizbil, 13.4.1977” / “*Metadromius arabicus* n.sp. J. Mateu det. 1977”. [**NHMB**] (Fig. 26). Al Baha: 1♂, “KSA, Al Makhwa, Shada Al Aala, 19°50.411'N 41°18.686'E Alt. 1611 m, 27.I.2015, (LT)., 1♀, “19°51.066'N 41°18.037'E Alt. 1325 m, 27.I.2015, (LT)., 1♂, 2♀, “19°50.710'N 41°18.267'E Alt. 1474 m, 15.II.2014, (LT)., 3♂, 5♀, “19°50.575'N 41°18.691'E Alt. 1666 m, 02.III.2015, (LT)., 1♂, “19°51.066'N 41°18.037'E Alt.1325 m, 02.III.2015, (LT)., 1♂, 1♀, “19°51.762'N 41°18.089'E Alt. 1225 m, 02.III.2015, (LT), H. Al Dhafer, M.S. Abdel-Dayem, H. H. Fadl, A. El Turkey, A. Elgarbway & I. Rasool”. 1♀, “KSA, Al Baha, Wadi Turaba, 20°14.369'N 41°15.234'E Alt. 1757 m, 9.III.2012, (LT), M.S. Abdel-Dayem”. 51♂, 71♀ “19°50.329'N 41°18.604'E Alt. 1563 m, 02.IX.2015, (LT)., 2♂, ♀, “19°51.066'N 41°18.037'E Alt. 1325 m, 02.IX.2015, (LT)., 14♂, 22♀, “19°50.575'N 41°18.691'E Alt. 1666 m, 02.IX.2015, (LT)., 37♂, 51♀, “19°50.710'N 41°18.267'E Alt. 1474 m, 02.IX.2015, (LT)., 2♀, “19°51.762'N 41°18.089'E Alt. 1225 m, 02.IX.2015, (LT)., 88♂, 96♀, “19°50.411'N 41°18.686'E Alt. 1611 m, 02.IX.2015, (LT)., Al Dafer H., M.S. Abdel Dayem., H. H. Fadl., El Gharbawy., El Turkey & Soliman, A”. 3♂, 6♀, “19°50.329'N 41°18.604'E Alt. 1563 m, 17.X.2014, (LT)., 1♂, 2♀, “19°51.762'N 41°18.089'E Alt. 1225 m, 17.X.2014, (LT)., 3♂, 2♀, “19°50.710N’ 41°18.267'E Alt. 1474 m, 17.X.2014, (LT)., 1♂, 1♀, “19°51.066'N 41°18.037'E Alt. 1325 m, 17.X.2014, (LT)., 4♂, 2♀, “19°50.411'N 41°18.686'E Alt. 1611 m, 17.X.2014, (LT), H. Al Dhafer, M.S. Abdel-Dayem, H. H. Fadl, A. El Turkey, A. Elgarbway & I. Rasool”. 1♀, “19°52.598'N 41°18.672'E Alt. 892 m, 17. X.2014, (LT)., 1♀, “19°50.391'N 41°18.634'E Alt. 1562 m, 03.XI.2013, (HP), I. Rasool”. 1♂, “19°52.685'N 41°18.663'E Alt. 851 m, 14.XI.2015, (LT)., 2♂, 2♀, “15.XI.2015, (LT)., 2♀, “19°50.329'N 41°18.604'E Alt. 1563 m, 18.XI.2015, (LT)., 1♂, 1♀, “19°52.598'N 41°18.672'E Alt. 892 m, 15.XI.2015, (LT)., 3♂, 5♀, “19°51.066'N 41°18.037'E Alt. 1325 m, 14.XI.2015, (LT)., 2♂, “19°50.329'N 41°18.604'E Alt. 1563 m, 14.XI.2015, (LT)., 5♂, 3♀, “19°52.717'N 41°18.712'E Alt. 825 m, 15.XI.2015, (LT)., 2♂, 4♀, “13.XI.2015, (LT), Al Dafer H., M.S. Abdel Dayem., H. H. Fadl., El Gharbawy., El Turkey & Soliman, A”. 1♀, “KSA, Wadi Saad Dam, 20°07.605'N 41°21.459'E (HP), I. Rasool”. Asir: 4♀, “KSA, Abha, Rayda, 18°11.749'N 42°23.345'E Alt.1614 m, 21.II.2014, (LT)., 2♂, 4♀, “18°11.679'N 42°23.691'E Alt. 1851 m, 21.II.2014, (LT)., 5♂, 5♀, “18°11.618'N 42°23.42'E Alt. 1772 m, 21.II.2014, (LT), H. Al Dhafer, M.S. Abdel-Dayem, H. H. Fadl, A. El Turkey, A. Elgarbway & I. Rasool”. 1♂, 2♀, “Wadi Maraba, 18°19.79'N 42°40.952'E Alt. 1467 m, 23.II.2014, (HP), Rasool, I”. 3♂, 7♀, “Wadi Rayda, 18°11.749'N 42°23.345'E Alt.1614 m, 24.II.2014, (LT), Rasool, I”. 6♂, 9♀, “18°11.749'N 42°23.345'E Alt. 1614 m, 24.III.2014, (LT), S. Soonbati”. 1♂, 1♀, “18°11.884'N 42°24.435'E Alt. 2387 m, 04.III.2015, (LT)., 1♀, “18°11.766'N 42°24.315'E Alt. 2285 m, 04.III.2015, (LT)., 2♂, “18°11.695'N 42°23.818'E Alt. 1897 m, 04.III.2015, (LT)., 5♂, 7♀, “18°11.679'N 18°11.679'E Alt. 1851 m, 04.2015, (LT)., 3♂, 1♀, “18°11.618'N 42°23.42'E Alt. 1772 m, 04.III.2015, (LT), Al Dafer H., M.S. Abdel Dayem., H. H. Fadl., El Gharbawy., El Turkey & Soliman, A”. 1♂, 2♀, “18°11.695'N 42°23.818'E Alt. 1897 m, 26.IV.2014, (LT)., 1♂, “18°11.679'N 42°23.691'E Alt. 1851 m, 06.VI.2014, (LT)., 3♂, 6♀, “18°11.618'N 42°23.42'E Alt. 1772 m, 26.VIII.2014, (LT)., 1♀, “18°11.695'N 42°23.818'E Alt. 1897 m, 26.VIII.2014, (LT)., 3♀, “18°11.618'N 42°23.42'E Alt. 1772 m, 20.X.2014, (LT)., 1♂, 1♀, “18°11.679'N 18°11.679'E Alt. 1851 m, (LT)., 2♂, “18°11.749'N 42°23.345'E Alt. 1614 m, (LT)., 1♂, 3♀, “18°11.695'N 42°23.818'E Alt. 1897 m, (LT)., 2♂, 1♀, “18°11.766'N 42°24.315'E Alt. 2285 m, (LT)., 4♂, 2♀, “18°11.884'N 42°24.435'E Alt. 2387 m, (LT), H. Al Dhafer, M.S. Abdel-Dayem, H. H. Fadl, A. El Turkey, A. Elgarbway & I. Rasool”. 1♂, “Al Magardah, Wadi Yabah, 19°14.911'N 41°47.255'E Alt. 402 m, 11. X.2013, (HP), I. Rasool, M. Al Harbi, S. Soonbati & S. Khan”. 1♀, “18°11.749'N 42°23.345'E Alt. 1614 m, 17.XI.2015, (LT)., 1♂, “17.XI.2015, (HP)., 1♀, “18°12.315'N 42°24.607'E Alt. 2578 m, 18.XI.2015, (LT)., 1♂, 3♀, “18°11.679'N 18°11.679'E Alt. 1851, 18.XI.2015, (LT)., 2♂, 2♀, “18°11.618'N 42°23.42'E Alt. 1772 m, 18.XI.2015, (LT)., 1♂, “18°11.884'N 42°24.435'E Alt. 2387 m, 18.XI.2015, (LT)., 1♂, 1♀, “18°11.695'N 42°23.818'E Alt. 1897 m, 18.XI.2015, (LT), Al Dafer H., M.S. Abdel Dayem., H. H. Fadl., El Gharbawy., El Turkey & Soliman, A”. 1♂, “18°11.766'N 42°24.315'E Alt. 2285 m, 12.XII.2014, (LT)., 2♂, “18°11.695'N 42°23.818'E Alt. 1897 m, (LT)., 1♂, 3♀, “18°11.679'N 18°11.679'E Alt. 1851 m, (LT)”. 1♂, 2♀, “18°11.749'N 42°23.345'E Alt. 1614 m, (LT)., 1♀, “18°11.618'N 42°23.42'E Alt. 1772 m, (LT), H. Al Dhafer, M.S. Abdel-Dayem, H. H. Fadl, A. El Turkey, A. Elgarbway & I. Rasool” [**KSMA].**

##### Description.

Small beetles (Fig. 25) TBL 2.43–3.00 mm. *Color*: Frons and vertex dark brown or rufo-testaceous; neck, mouthparts, antennae, pronotum, elytra, epipleurae and legs testaceous; antennae, mouthparts and legs sometimes pale testaceous; abdomen pale testaceous; elytra with dark brown transverse band at middle, not reaching to lateral margin and apex, prolonged along suture to the base of elytra. *Microsculpture*: Labrum with isodiametric transverse pattern; head without microsculpture; last sternum of abdomen sometimes with depressed microlines. *Head*: as long as wide HL 0.52–0.66 mm and HW 0.56–0.63 mm, narrower than pronotum; dorsum finely and sparsely punctuated (Fig. 14). *Pronotum*: Transverse, PW 0.61–0.66 mm PL 0.42–0.49 mm, narrowed posteriorly with sharp basal angles, base incised near the angles (Fig. 14); dorsum finely punctuated. *Elytra*: Subparallel, EL 1.42–1.73 mm, EW 0.99–1.20 mm; widest behind the middle; apex obliquely truncate. Claws weakly dentate. *Abdomen*: All sternites smooth, except last sternum with short and fine pubescence; apical margins of last sternum rounded and tetra–setose in females, slightly incised in middle and bi–setose in males. *Aedeagus*: Elongate (Fig. 40), AL 0.53 mm; in lateral view it is curved dorsally, narrowed at both ends, thick and broad before apical lamina; apical lamina elongate, rounded at the end; endophallus armature almost rounded

##### Affinities.

This species is very close to *Metadromius* spec. (see below) in general appearance, body form, shape of head, pronotum, and elytra, but can be easily distinguished by its testaceous pronotum, smooth head, transverse band on elytra, and short apical lamina of aedeagus, with an elongate endophallus armature.

##### Ecological notes.

It is collected from low lands to high lands in diverse habitats within 402–2387 m range of altitude (Fig. 51). It is found under stones among vegetation and in sandy ranges, which are influenced by rain water. The species was collected during all months of the year except July.

##### Geographical distribution.

This species is described from Saudi Arabia (Mateu 1979) and distributed in southwest Saudi Arabia, also reported from Iran and United Arab Emirates (Felix 2009, Kabak 2017). It is W–Palaearctic element that exemplifies SW–Asiatic chorotype.

#### Metadromius brittoni

Taxon classificationAnimaliaColeopteraCarabidae

(Basilewsky, 1948)

Figures 7 , 15 , 27 , 41 , 51

Philorhizus brittoni Basilewsky, 1948: 129.

##### Type locality.

Yemen, Dahla

##### Type depository.

Holotype in BMNH

##### Material examined.

Total 1,362 specimens: Al Baha: 1♂, “KSA, Al Makhwa, Shada Al Aala, 19°51.762'N 41°18.089'E Alt. 1225 m, 27. I.2015, (LT)., 1♀, “19°52.598'N 41°18.672'E Alt. 892 m, 16.II.2014, (LT), M.S. Abdel-Dayem & I. Rasool”. 1♂, 1♀, “19°50.329N’ 41°18.604'E Alt. 1563 m, 21.IV.2014, (LT)., 2♂, 3♀, “19°51.066'N 41°18.037'E Alt. 1325 m, 21.IV.2014, (LT)., 17♂, 9♀, “19°51.762'N 41°18.089'E Alt. 1225 m, 21.IV.2014, (LT)., 1♀, “19°50.710'N 41°18.267'E Alt. 1474 m, 21.IV.2014, (LT)., 12♂, 8♀ “19°52.598'N 41°18.672'E Alt. 892 m, 23.IV.2014, (LT)., 21♂, 16♀, “19°51.762'N 41°18.089'E Alt. 1225 m, 03.VI.2014, (LT)., 8♂, 5♀, “19°51.762'N 41°18.089'E Alt. 1225 m, 23.VIII.2014, (LT)., 13♂, 9♀, 19°51.066'N 41°18.037'E Alt. 1325 m, 23.VIII.2014, (LT)., 1♂, 19°50.710'N 41°18.267'E Alt. 1474 m, 23.VIII.2014, (LT)., 1♂,, “19°50.411'N 41°18.686'E Alt. 1611 m 23.VIII.2014, (Sucking), H. Al Dhafer, M.S. Abdel-Dayem, H. H. Fadl, A. El Turkey, A. Elgrbaway & I. Rasool”. 2♂, 4♀, “19°51.066'N 41°18.037'E Alt. 1325 m, 16.X.2014, (LT), I. Rasool”. 24♂, 14♀, “19°52.598'N 41°18.672'E Alt. 892 m 17.X.2014, (LT), I. Rasool & M. Al Harbi”. 3♀, “19°51.762'N 41°18.089'E Alt. 1225 m, 17. X.2014, (LT)., 1♀, “19°51.762'N 41°18.089'E Alt. 1225 m, 18. X.2014, (PT)., 2♂, 5♀, “19°51.066'N 41°18.037'E Alt. 1325 m, 17. X.2014, (LT)., 1♂, “19°50.710'N 41°18.267'E Alt. 1474 m, (LT)., “19°50.411'N 41°18.686'E Alt. 1611 m, (LT)., 1♀, “19°50.411'N 41°18.686'E Alt. 1611 m, 18. X.2014, (PT)., 1♂, “19°50.391'N 41°18.634'E Alt. 1562 m, 03.XI.2013, (HP), I. Rasool”. 1♀, “Wadi Neera, 19°44.870'N 41°20.008'E Alt. 471 m, 10.XII.2014, (LT), H. Al Dhafer, M.S. Abdel-Dayem, H. H. Fadl, A. El Turkey, A. Elgarbway & I. Rasool”. 1♀, “Al Mandaq, Wadi Turbah, 20°12.937'N 41°17.176'E Alt. 1793 m, 10.V.2011, (HP), M.R. Sharaf”. 4♂, 2♀, Wadi Saad, 20°07.605'N 41°21.459'E 17.X.2014, (HP), I. Rasool”. Asir: 2♂, 2♀, “KSA, Abha, Wadi Maraba, 18°19.79'N 42°40.952'E Alt. 1467 m, 23.II.2014, (HP)., 1♂, “Wadi Rida, 18°11.749'N 42°23.345'E Alt. 1614 m, 24.II.2014, (LT), I. Rasool”. 6♂, 21♀, “Wadi Rida, 18°11.749'N 42°23.345'E Alt. 1614 m, 24.III.2014, (LT), S. A. El-Sonbati”. 2♂, “Rayda, 18°11.766'N 42°24.315'E Alt. 2285 m, 26.IV.2014, (LT)., 26♂, 28♀, “18°11.749'N 42°23.345'E Alt. 1614 m, 26.IV.2014, (LT)., 1♀, “18°11.679'N 18°11.679'E Alt. 1851 m, (LT)., 1♂, 2♀, “18°11.695'N 42°23.818'E Alt. 1897 m, 26.IV.2014, (LT)., 1♀, “18°11.618'N 42°23.42'E Alt. 1772 m, 26.VIII.2014, (LT)., 1♂, 2♀, “18°11.749'N 42°23.345'E Alt.1614 m, 20.X.2014, (LT)., 1♂, “18°11.679'N 42°23.691'E Alt. 1851 m, 06.VI.2014, (LT), H. Al Dhafer, M.S. Abdel-Dayem, H. H. Fadl, A. El Turkey, A. Elgarbway & I. Rasool”. 509♂ 455♀, “Al Hubail, Wadi Reem, 18°06.981'N 42°13.939'E Alt. 451 m, 20.X.2014, (LT), I. Rasool & M. Al Harbi”. 20♂, 14♀, “Al Magardha, Wadi Yabah, 19°14.911'N 41°47.255'E Alt. 402 m, 11. X.2013, (LT), I. Rasool, M. Al Harbi, S. Soonbati & S. Khan”. 5♂, 3♀, “Rayda 18°11.766'N 42°24.315'E Alt. 2285 m, 11.XII.2014, (LT), H. Al Dhafer, M.S. Abdel-Dayem, H. H. Fadl, A. El Turkey, A. Elgarbway & I. Rasool”. Jazan: 1♀, “KSA, Fifa, Al Absia, 17°15.831'N 43°60.498'E Alt. 1770 m, 20.III.2014, (LT), 30♂, 12♀ “17°15.831'N 43°60.498'E Alt. 1770 m, 23.III.2014, (LT)., 2♀, “Jazan Road, 17°20.223'N 43°07.539'E 1770 m, 21.III.2014, (LT)., 1♀, “Fifa, Agricultural research station, 17°28.671'N 43°14.39'E Alt. 879 m, 06.IV.2013, (HP), M.R. Sharaf” [**KSMA].**

##### Description.

Small beetle (Fig. 27) TBL 2.13–2.75 mm. *Color*: Head, lateral margin of abdominal sternite and posterior half of epipleurae dark brown; pronotum, elytra, anterior half of epipleurea and antennae testaceous; legs, ventrum of thorax and abdominal sternite pale testaceous; elytra with dark brown pattern covering the posterior half of elytra, leaving the testaceous round spots near apex and suture, suture dark brown throughout. *Microsculpture*: head, clypeus, labrum, pronotum, and elytra with mesh isodiametric pattern; last abdominal sternum with transverse microlines, rest of the sternites with depressed microlines. *Head*: As long as wide, narrower than pronotum; HL 0.45–0.52 mm and HW 0.46–0.52 mm; dorsum densely and coarsely punctate (Fig. 15). *Pronotum*: Transverse, PW 0.55–0.58 mm and PL 0.40–0.44 mm, pronotum narrowed posteriorly with sharp basal angles, base lobate at middle, incised near the angles; dorsum of pronotum densely and coarsely punctate (Fig. 15). *Elytra*: Subparallel, slightly widened posteriorly, EL 1.34 mm EW 0.95 mm apical margin obliquely truncate, sparsely punctuate, claws dentate. *Abdomen*: All visible sternite sparsely and finely punctate; last sternum Tetra–setose, incised in males and rounded in females. *Aedeagus*: It is elongate (Fig. 41), AL 0.45 mm; in lateral view flat throughout, very thin and equally broadened from base to apical end; apical lamina broadened, end strongly rounded.

##### Affinities.

This species is similar to *M. arabicus* and *M. ephippiatus* in general from but it can be differentiated by: densely punctated head and pronotum, presence of microsculptures on whole body, Tetra–setose apical margin of abdominal sternum in male, and apical lamina of aedeagus broad.

##### Ecological notes.

This species is attracted to UV–light. Living from low to high elevated areas from 402–2761 m (Fig. 51). It is collected from various kind of habitats, sand dunes covered with light vegetation, near the water streams and under the shade of small shrubs where it lives with variety of arthropods (Hemipetra, Collembola, Staphylinidae, and spiders), while in valleys of mountains, it is hidden under leaves and stones.

##### Geographical distribution.

It was described from Yemen (Basilewsky 1948), then recorded from Jordan and Saudi Arabia (Mateu 1979, Kabak 2003, 2017, Anichtchenko 2017). *Metadromius brittoni* is Arabian element that exemplifies Arabian chorotype.

#### Metadromius

Taxon classificationAnimaliaColeopteraCarabidae

spec.

Figures 28 , 51

##### Material examined.

Total seven specimens: One female labeled as, “Saudi Arabien, W. Büttiker” / “W. Shuqub Turabah, 1250 m, 21.IV.1980”/ “*Metadromius ephippiatus*, Fairm., J. Mateu, det. 1983” [**NHMB].** Al Baha: 1♂, “KSA, Al Baha, Al Makhwa, Wadi Neera, 19°44.870'N 41°20.008'E Alt. 471 m, 3.III.2015, (LT)., 1♀, Shada Al Aala “19°51.066'N 41°18.037'E Alt. 1325 m, 2.III.2015, (LT), H. Al Dhafer, M.S. Abdel-Dayem, H. H. Fadl, A. El Turkey, A. Elgarbway & I. Rasool”. Asir: 2♂, 2♀, “KSA, Al Magardah, Wadi Yabah, 19°14.911'N 41°47.255'E Alt. 402 m, 11.X.2013, (LT), I. Rasool, M. Al Harbi, S. Soonbati & S. Khan” [**KSMA].**

##### Notes.

These specimens (Figs 28) are similar to specimens identified by Mateu (1986) from Saudi Arabia as *M. ephippiatus* (Fairmaire, 1884). However, the specimens of *M. ephippiatus* known from Algeria are quite different and identification of Mateu, 1986 is doubtful. Most probably the specimens from Saudi Arabia are a new species. As *Metadromius* in the Middle East is under revision, a species name is not designated here.

#### Microlestes

Taxon classificationAnimaliaColeopteraCarabidae

Schmidt-Gobel, 1846

##### Type species.

*Microlestes inconspicuus* Schmidt-Göbel, 1846

The genus *Microlestes* is the largest genus of Dromiusina encompass about 130 species all over the world (Lorenz 2005, Anichtchenko 2017), distributed in Palaearctic (Middle East and Asia), Nearctic, Afrotropical, Oriental and Neotropical regions (Mateu 1971, Bousquet 2012). Kabak (2003, 2017) in his catalogue, mentioned 63 *Microlestes* species from Palaearctic region. The members of *Microlestes* can be distinguished from other related genera in Dromiusina by the combination of the characters: small sized beetle ranges from 2.30–3.40 mm; labrum truncate at anterior margins; mentum without median tooth; pubescence of antennae starts from antennomeres III; lateral margins of pronotum sinuated posteriorly, base of pronotum weakly incised and curved towards hind angles and rounded in the middle; basal tarsomere of hind legs distinctly longer than last, claws sparsely dentate; elytra parallel sided and transversally truncated at apex (Lindroth 1974, Bousquet 2010). So far eight species have been known from Saudi Arabia (Mateu 1979, 1986, Kabak 2003, 2017). Only two species *Microlestes infuscatus fragilis* Mateu, 1956 and *Microlestes glabrellus* (Reitter 1901) are occuring in the southwestern part of the country (Mateu 1986). In the present study, *Microlestes discoidalis* (Fairmaire 1892) is also newly recorded from the southwest of Saudi Arabia.

#### Microlestes discoidalis

Taxon classificationAnimaliaColeopteraCarabidae

(Fairmaire, 1892)

Figures 16 , 29 , 42 , 52

Blechrus discoidalis Fairmaire, 1892: 83.
Microlestes schmiedeknechti Pic, 1900: 91.

##### Type locality.

Djibouti, Obock

##### Type depository.

Holotype in MHNP.

##### Material examined.

51 specimens: Al Baha: 2♂, 1♀, “KSA, Al Makhwa, Shada Al Aala, 19°52.598'N 41°18.672'E Alt. 892 m, 26. I.2015, (LT)., 3♂, 8♀, “19°52.598'N 41°18.672'E Alt. 892 m, 15-16. II.2014, (LT), I. Rasool, 1♂ “19°50.329'N 41°18.604'E Alt. 1563 m, 21. IV.2014, (LT)., 1♂, “19°52.598'N 41°18.672'E Alt. 892 m, 23.IV.2014, (LT), H. Al Dhafer, M.S. Abdel-Dayem & H. H. Fadl, I. Rasool”. 1♂, 1♀, “19°52.685'N 41°18.663'E Alt. 851 m, 15.XI.2015, (LT)., 1♀, “19°52.717'N 41°18.712'E Alt. 825 m, 13.XI.2015, (LT), Al Dafer H., M.S. Abdel-Dayem., H. H. Fadl., El Gharbawy., El Turkey & Soliman, A”. Asir: 1♀, “KSA, Abha, Rayda, 18°11.749'N 42°23.345'E Alt. 1614 m, 24.II.2014, (LT), I. Rasool”. 1♂, “Wadi Rayda, 18°11.749'N 42°23.345'E Alt. 1614 m, 24.III.2014, (LT), S. A. El-Sonmbati”. 1♀, “18°11.749'N 42°23.345'E Alt. 1614 m, 30. I.2015, (LT), H. Al Dhafer, M.S. Abdel-Dayem, H. H. Fadl, A. El Turkey, A. Elgarbway & I. Rasool”. 2♀, “Al Manznar, Wadi Baqrah, 18°47.476'N 41°56.310'E Alt. 331 m, 13.III.2012, (LT), H. AL Dhafer, M. S Abdel-Dayem & H. H. Fadl”. 1♂, “Wadi Quonunah, 19°25.457'N 41°36.141'E Alt. 353 m, 12.V.2011, (LT), M.R. Sharaf”. 1♀, “Al Magardah, Wadi Yabah, 18°47.977'N 42°01.375'E Alt. 411 m, 2.VI.2012, (LT), H. Al Dhafer & A. Al Ansi”. 2♀, “19°14.911'N 41°47.255'E Alt. 402 m, 11.X.2013, (LT)., 5♀, 1♂, “Al Hubail, Wadi Reem, 20.X.2014, 18°06.981'N 42°13.939'E Alt. 451 m, (LT), I. Rasool & M. Al Harbi”. Jazan: 6♂, 7♀, “KSA Adrab, Wadi Baiz, 17°37.562'N 42°22.242'E Alt. 75 m, 24.II.2015, (HP), I. Rasool”.1♂, 2♀, “Fifa, Al Absia, 17°15.831'N 43°60.498'E Alt. 1770 m, 23.III.2014, (LT)., 1♂, “17°15.831'N 43°60.498'E Alt. 1770 m 20.III.2014, (LT), S. A. El-Sonmbati” [**KSMA].**

##### Description.

Small beetles (Fig. 29), TBL 2.25–3.47 mm. *Color*: Dorsum and ventrum of head and pronotum black; femora, tarsomeres, mouthparts and epipleurae dark brown; antennae, abdomen, lateral margins, suture, base and apex of elytra -dark brown, elytra with two large pale testaceous macula. *Microsculpture*: Head, clypeus and labrum with isodiametric mesh pattern; pronotum with irregular and ventrum of head, thorax and abdomen with regular transverse microlines; elytra with transverse microlines on base, apex and lateral margins. *Head*: as long as wide, as wide as width of pronotum, HL and HW 0.52–0.70 mm; eyes large and prominent, tempora short (Fig. 16) *Pronotum*: Slightly wider than long, PL 0.38–0.56 mm and PW 0.49–0.69 mm; pronotum narrowed and sinuate posteriorly, basal angles very weak, base of pronotum lobate in middle (Fig. 16). *Elytra*: Elytra parallel sized; EL 1.07–1.77 mm, EW 0.78–1.27 mm. *Abdomen*: Apical margin of last sternum bi–setose in both males and females, rounded in males, slightly incised in females. *Aedeagus*: Shape of aedeagus (Fig. 42), AL 0.58 mm; in lateral view, aedeagus strongly curved dorsally and ventrally, blunt base, broadened in the middle, narrowed apically; apical lamina elongated and with blunt end; endophallus armature of aedeagus elongate and slender with pointed end.

##### Affinities.

This species is very similar to *M. glabrellus* (Reitter 1901) in body size, shape of pronotum, large eyes, and short tempora, but can be differentiated by the two large and elongated pale testaceous discal maculae on the elytra and suppressed transverse microlines on the elytra. Endophallus armature of aedeagus elongate and slender.

##### Ecological notes.

The species was attracted to UV–light at low elevated areas to high mountainous areas at 75–1770 m range of altitude (Fig. 52). In day time, it remains hidden under gravels and leaf litter below the shade of small shrubs and vegetation; and can be easily collected by aspirator.

##### Geographical distribution.

This species was described from Djibouti (Fairmaire 1892) and is now widely distributed in Afghanistan, Chad, Eritrea, Iran, Israel, Kenya, Mauritania, Niger, Oman, Saudi Arabia, Somalia, Sudan, Turkey, Yemen, United Arab Emirates (Felix 2009, Kabak 2003, 2017, Anichtchenko 2017). It exemplifies Afrotropico–Indo–Mediterranean chorotype.

#### Microlestes glabrellus

Taxon classificationAnimaliaColeopteraCarabidae

(Reitter, 1901)

Figures 17 , 30 , 43 , 52

Blechrus glabrellus Reitter, 1901: 380.
Microlestes arabicus Mateu, 1956.
Microlestes flavipes Holdhaus, 1912.

##### Type locality.

Egypt.

##### Type depository.

Holotype in MNH.

##### Material Examined.

Total 7 specimens: Asir: 1♀, “KSA, Abha, Rayda, “18°11.749'N 42°23.345'E Alt. 1614 m, 30. I.2015, (LT)., 1♀, “4.III.2015, (LT)., 1♀, “18°11.695'N 42°23.818'E Alt. 1897 m, 31.VII.2014, (LT)., 1♂, “18°11.749'N 42°23.345'E Alt. 1614 m, 26. VIII.2014, (LT)., 1♂ “18°11.618'N 42°23.42'E Alt. 1772 m, 26.VIII.2014, (LT), H. Al Dhafer, M.S. Abdel-Dayem, H. H. Fadl, A. El Turkey, A. Elgarbway & I. Rasool”. 1♂, 1♀, “18 10.293'N 42 22.195'E Alt. 1150 m, 16.IV.2016, (LT), H. Al Dhafer, M.S. Abdel-Dayem, B. Daniele, A. Al Ansi, A. Soliman & I. Rasool” [**KSMA**].

##### Description.

Subparallel sized beetle (Fig. 30), TBL 3.12–3.60 mm. *Color*: Overall black, dorsum and ventrum of head, pronotum and abdomen, mouthparts and femora black; antennae, epipleurae, tibiae and tarsomeres -dark brown; coxae dark brown; elytra bronze to black. *Microsculpture*: Head along with clypeus and labrum with isodiametric mesh pattern; elytra, epipleurae, ventrum of thorax and abdominal sternite with transverse microlines. *Head*: As long as wide, almost as wide as pronotum, HL and HW 0.65–0.71 mm; eyes large, tempora short (Fig. 17). *Pronotum*: wider than long, PL 0.49–0.56 mm, PW 0.68–0.75 mm; pronotum narrowed posteriorly with weak basal angles; base of pronotum lobed (Fig. 17). *Elytra*: Subparallel sized, EL 1.61–1.77 mm EW 1.15–1.21 mm; apex of elytra transversally truncates; claws weakly dentate. *Abdomen*: apex of last abdominal sternum bi–setose and rounded in both males and females; *Aedeagus*: Shape of aedeagus (Fig. 43), AL 0.56 mm, in lateral view, aedeagus curved dorsally, straight in the middle ventrally, broad from the base to apical lamina; apical plat long narrowed with elongate and blunt end; internal sac broad and flat.

##### Affinities.

This species is similar to *M. discoidalis* in general appearance, elongate and slender, antennae, eyes large with short temples, but can be distinguished by black color of whole body (except tibiae and tarsomeres), strong transverse microlines on elytra, and endophallus armature of aedeagus broad and flat.

##### Ecological notes.

Members of this species were found in steep slopes in Rayda Nature Reserve (Asir Provence). They were collected from 1614–1897 m of elevation (Fig. 52), adult beetles were fly to UV-light. The adults were collected from places covered by different vegetation that dominated by Cactus shrubs *Opuntiaficus indica*.

##### Geographical distribution.

This species was described from Egypt (Reitter 1901), and then reported from Chad, Ethiopia, Mauritania, Niger, Senegal, Saudi Arabia, and Yemen (Mateu 1986, Kabak 2003, 2017, Anichtchenko 2017). Its geographical range exemplifies Saharo – Sahelo – Arabian Chorotype.

#### Microlestes infuscatus fragilis

Taxon classificationAnimaliaColeopteraCarabidae

Mateu, 1956

**Figures 31–36.**
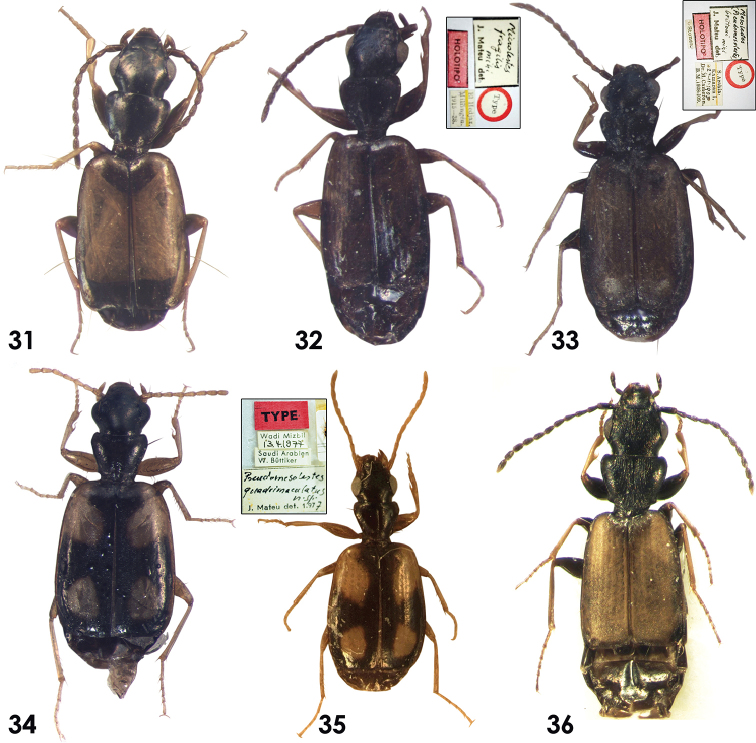
Habitus of Dromiusina species: **31, 32** *Miceolestes infuscatus fragilis* Mateu, 1956 **33** *Pseudomesolestes brittoni* Mateu, 1956 **34, 35** *P. quadriguttatus* Matue, 1979 **36** *Zolotarevskyella rhytidera* (Chaudoir, 1876).

Figures 18 , 31 , 32 , 44 , 52

##### Type locality.

Saudi Arabia, Hejaz.

##### Type depository.

Holotype in BMNH.

##### Material examined.

Total 55 specimens: HOLOTYPE: Male labeled “Hedjaz, Millingen, 1915-38” / “Holotype [red square label]” / “*Microlestes fragilis*, J. Mateu, det.” / “Holotype [red round label]”. [**BMNH**] (Fig. 32). Al Baha: 1♂, 2♀, “KSA, Al Makhwa, Shada Al Aala, 19°52.598'N 41°18.672'E Alt. 892 m, 26.I.2015, (LT), I. Rasool”. 3♂, 5♀, “Thee Ain Village, 19°55.774'N 41°26.574'E Alt. 754 m, 10.III.2012, (HP), M.S. Abdel-Dayem”. 2♂, 3♀, “19°55.465'N 41°26.343'E Alt. 744 m, 07.IV.2013, (HP), M.R. Sharaf”. 7♂, 9♀, “19°52.598'N 41°18.672'E Alt. 892 m, 23.IV.2014, (LT), H. Al Dhafer, M.S. Abdel-Dayem, H. H. Fadl, A. El Turkey, A. Elgarbway & I. Rasool”. 2♀, “9°55.459'N 41°26.302'E Alt. 741 m, 11.V.2013, (HP), M.R. Sharaf”. 2♂, 3♀, “Al Mandaq, Wadi Turba, 20°12.937'N 41°17.176'E Alt. 1793 m, 14.V.2011, (HP)., 2♂, “Wad Elzaraeb, 20°04.243'N 41°23.123'E Alt. 2086, (HP), M.R. Sharaf”. 1♂, “19°51.066'N 41°18.037'E Alt. 1325 m, 23.VIII.2014 (LT), H. Al Dhafer, M.S. Abdel-Dayem, H. H. Fadl, A. El Turkey, A. Elgarbway & I. Rasool”. 1♀, “19°50.710'N 41°18.267'E Alt. 1474 m, 02.IX.2015, (LT)., 1♂, “19°50.575'N 41°18.691'E Alt. 1666 m, 02.XI.2015, (LT), H. Al Dhafer, M.S. Abdel-Dayem, H. H. Fadl, A. El Turkey, A. Elgarbway & I. Rasool”. 1♀, “19°50.575'N 41°18.691'E Alt. 1666 m, 15.XI.2015, (LT), H. Al Dhafer, M.S. Abdel-Dayem, H. H. Fadl, A. El Turkey, A. Elgarbway & A. Soliman”. Asir: 1♂, “KSA, Abha, Rayda, “18°11.695'N 42°23.818'E Alt. 1897 m, 26.IV.2014, (LT)., 2♀, “18°11.766'N 42°24.315'E Alt. 2285 m, 8.VI.2014, (PT), H. Al Dhafer, M.S. Abdel-Dayem, H. H. Fadl, A. El Turkey, A. Elgarbway & I. Rasool”. 2♂, 1♀, “Al Magardah, Wadi Yabah, 19°14.911'N 41°47.255'E Alt. 402 m, 11.X.2013, (LT)., 1♂, 1♀, “Wadi Talalea, 19°2.74'N 41°46.333'E Alt. 259 m, 12.X.2013, (LT), I. Rasool, M. A. Al Mushairi, S. Sonmbaati, S. Khan”. Jazan: 2♂, 2♀, “Fayfa, Dayer Beni, 17°28.797'N 43°.14.434'E Alt. 871 m, 4.IV.2013, (HP), M. R. Sharaf” [**KSMA**].

##### Description.

Small beetle (Fig. 31), TBL 2.27–2.55 mm. *Color*: Head and pronotum black, pronotum sometimes–dark brown; sub scutellum, apical forth of elytra, epipleurae, femora, thoracic and abdominal ventrum, antennae, mouthparts and labrum–dark brown; rest of the elytra, tibiae and tarsomeres ferruginous. *Microsculpture*: Head with longitudinal microlines; clypeus with isodiametric mesh pattern; labrum, pronotum, elytra, thoracic and abdominal ventrum with transverse microlines; irregular on abdominal sternite. *Head*: As long as wide, HL 0.48–0.56 mm, HW 0.44–0.51 mm; eyes small with long tempora (Fig. 18). *Pronotum*: Transverse, almost as wide as head, PL 0.34–0.38 mm and PW 0.52–0.58 mm; pronotum narrowed posteriorly, sinuate before basal angles, base of pronotum lobate in the middle. *Elytra*: Parallel sized; EL 1.13–1.20 mm, EW 0.79–0.85 mm; apex transversally truncates, claws weakly dentate in the middle. *Abdomen*: apical margin of last sternum rounded in males and slightly incised in females. *Aedeagus*: small (Fig. 44), AL 0.38 mm; in lateral view, strongly curved; basal part up to apical lamina thick, widened in the middle, apical margin and lamina narrow and long; apical end small rounded; internal sacs leaf-like.

##### Affinities.

This species is very close to *Microlestes vittipennis* J.R Sahlberg, 1908 in general appearance, color, body size and pattern of elytra, but it can be distinguished by: antennomere II as long as III, eyes small with large temples, strongly curved, apical margin and lamina narrow and long; apical end small rounded; internal sacs leaf-like.

##### Ecological notes.

The species was collected from low lands and mountainous areas with 648–2285 m elevation range (Fig. 52). Adults were found under gravels and leaf litter in humid and moist places among mixed vegetation and shrubs. Also, they are attracted to UV–light.

##### Geographical distribution.

This species was described by Matue (1956) from southwestern Saudi Arabia and also reported from Afghanistan and Yemen (Mateu 1979, Kabak 2003). This range exemplifies Afrotropico–Indo–Mediterranean.

#### Pseudomesolestes

Taxon classificationAnimaliaColeopteraCarabidae

Mateu, 1956

##### Type species.

*Mesolestes brittoni* Mateu, 1956

The genus *Pseudomesolestes* is a small genus that contains only seven species, distributed in Palaearctic, Oriental, and Afrotropical regions (Anichtchenko 2017). Three species have been recorded from Palaearctic region, two of which are documented from Arabian Peninsula (Kabak 2003, 2017). This genus can be distinguished from other genera in subtribe Dromiusina by the following combination of characters: antennae stout, antennomeres II as long as III; pubescence starts from antennomeres II; mentum without median tooth; labrum rounded at anterior margins; maxillary palpi fusiform; pronotum constricted and sinuate posteriorly, base of pronotum straight in the middle, making 45° angle to hind angles; elytra broadened posteriorly, apex of elytra transversally truncates; basal boarder of elytra is complete up to scutellum (Mateu 1956, 1984). In Arabian Peninsula, this genus is represented by two species *P. brittoni* Mateu, 1956 described from Yemen and also recorded from Saudi Arabia, and *P. quadriguttatus* Matue, 1979 is endemic to Saudi Arabia only.

#### Pseudomesolestes brittoni

Taxon classificationAnimaliaColeopteraCarabidae

Mateu, 1956

Figures 19 , 33 , 45 , 53

Mesolestes brittoni Mateu, 1956: 66.

##### Type locality.

Yemen, Kamaran Island.

##### Type depository.

Male in BMNH.

##### Material examined.

Holotype: Male labeled “Holotype [red label]” / “stones” / “S. Arabia: Kamaran. I. 27-11-1903, Dr. M. Cameron. B.M. 1928-109” / “Mesolestes (Pseudomesolestes) brittoni, J. Mateu det.” / “Holotype [rounded label, red boarder]” [**BMNH**] (Fig. 33 in this work).

##### Description.

Small beetle (Fig. 33) 2.55 mm. *Color*: frons and vertex black; clypeus, labrum, dorsum and ventrum of head and thorax, mouthparts, elytra, antennomeres I and femora -dark brown; rest of the antennomeres dark brown; elytra with two pale testaceous elongate spots, one after humeri covering intervals IV–VI and second round small spot near apex of elytra, covering intervals IV and V; tibiae and tarsomeres pale testaceous. *Microsculpture*: Head, pronotum and elytra with granulated microsculptures, clypeus and labrum with transverse lines. *Head*: as long as wide HL 0.56 mm and HW 0.58 mm as wide as pronotum; tempora short (Fig. 19). *Pronotum*: Transverse, PL 0.42 mm and PW 0.58 mm, narrowed posteriorly, sinuate before the basal angles, base straight in the middle with weak angles (Fig. 19). *Elytra*: Widened posteriorly, apical margins transversally truncate; striae II with fine punctures. Claws smooth. *Aedeagus*: Small (Fig. 45) AL 0.61 mm, in lateral view, aedeagus slightly curved dorsally and ventrally; thick from base to apical lamina; apical lamina narrowed, short and slightly curved before end with a small tooth dorsally; base of aedeagus also with a small tooth.

##### Affinities.

*Pseudomesolestes quadriguttatus* is the only other specie recorded from Saudi Arabia which is close to *P. brittoni* in shape of head and pronotum, but can be distinguished by granulated microsculptures on head, pronotum and elytra, wrinkles on dorsum of pronotum along the medial impression; shape of testaceous spots; dark brown femora, aedeagus with single elongate endophallus armature and short apical lamina.

##### Geographical distribution.

This species was originally described from Yemen (Mateu 1956) and also recorded from Saudi Arabia (Mateu 1979, Kabak 2017). It is confined to Arabian Peninsula and exemplifies Arabian chorotype.

#### Pseudomesolestes quadriguttatus

Taxon classificationAnimaliaColeopteraCarabidae

Mateu, 1979

Figures 4 , 9 , 20 , 34 , 35 , 46 , 53

Pseudomesolestes quadriguttatus Matue, 1979: 148.

##### Type locality.

Saudi Arabia, Riyadh, Wadi Mizibl.

##### Type depository.

Holotype male in NHMB.**Material examined**. Total 18 specimens: **Holotype** (Fig. 35): Male labeled “Type [red label]” / “Saudi Arabien, W. Büttiker” / “Wadi Mizbil, 13.4.1977” / “*Pseudomesolestes quadriguttatus* n. sp J. Mateu det. 1977”. [**NHMB**]. Al Baha: 1♀, “KSA, Al Baha, Al Makhwa, Shada Al Aala, 19°52.598'N 41°18.672'E Alt. 892 m, 26.I.2015, (LT), I. Rasool”. 1♂, 19°51.066'N 41°18.037'E Alt. 1325 m, 2.III.2015, (LT), H. Al Dhafer, M.S. Abdel-Dayem, H. H. Fadl, A. El Turkey, A. Elgarbway, A. Al Ansi & I. Rasool”. 1♂, 2♀, “19°52.598'N 41°18.672'E Alt. 892 m, 18.X.2014, (LT), I. Rasool”. 1♀, 17.X.2014, (LT)., I. Rasool and M. Al Harbi”. 1♀, “19°50.329'N 41°18.604'E Alt. 1663 m, 17.X.2014, (LT), H. Al Dhafer, M.S. Abdel-Dayem, H. H. Fadl, A. El Turkey, A. Elgarbway & I. Rasool”. 2♀, “Raghadan, Wadi Saad dam, 20°07.605'N 41°21.459'E 17.X.2014 (LT)., 1♂, “Wadi Turaba 20°10.430'N 41°19.365'E 17.X.2014, (HP), I. Rasool”. 1♂, 1♀, “19°52.685'N 41°18.663'E Alt. 851 m, 15.XI.2015, (LT), H. Al Dhafer, M.S. Abdel-Dayem, H. H. Fadl, A. El Turkey, A. Elgarbway & A. Soliman”. Asir: 1♀ “KSA, Abha, Wadi Rayda, 18°11.749'N 42°23.345'E Alt. 1614 m, 24.II.2014, (LT), I. Rasool”. 1♂, “18°12.315'N 42°24.607'E Alt. 2761 m, 11.XII.2014, (LT), H. Al Dhafer, M.S. Abdel-Dayem, H. H. Fadl, A. El Turkey, A. Elgarbway & I. Rasool” [**KSMA].** 1♂, “KSA, Al Baha, Al Makhwa, Shada Al Aala, 19°52.598'N 41°18.672'E Alt. 892 m, 18.X.2014, I. Rasool” [**RMNH].**

**Figures 37–47.**
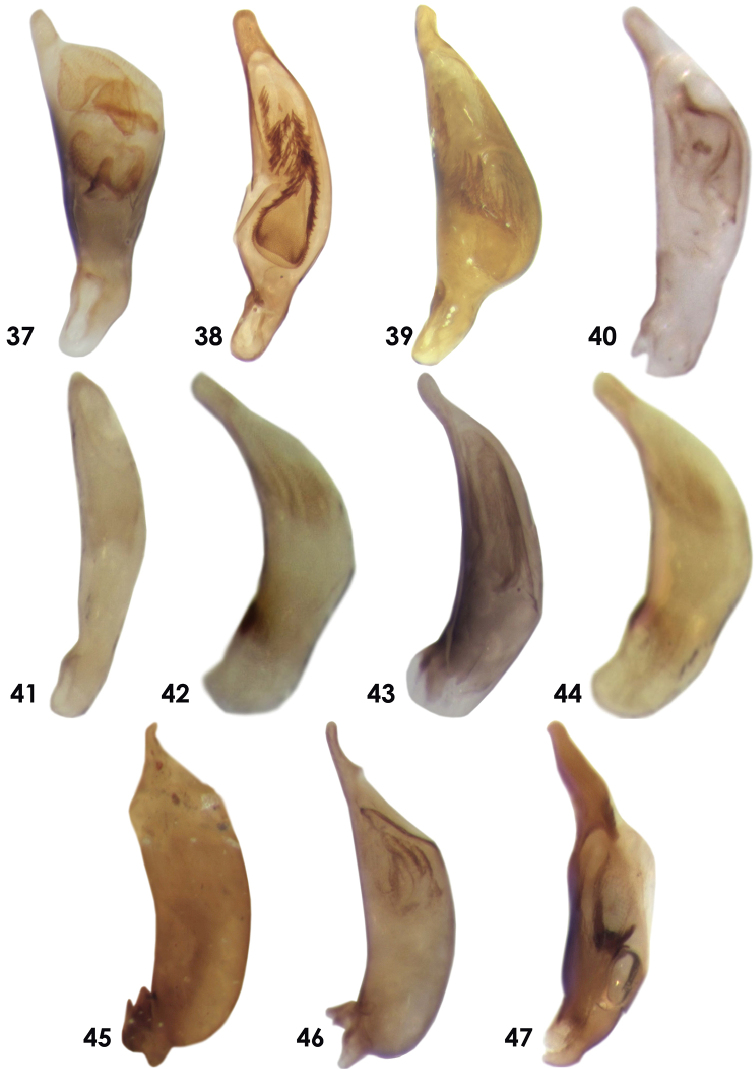
Aedeagus of Dromiusina species: **37** *Calodromius mayeti* (Bedel, 1907) **38** *Dromius saudiarabicus* sp. n. **39** *D. buettikeri* Mateu, 1990 **40** *Metadromius arabicus* Mateu, 1979 **41** *Metadromius brittoni* (Basilewsky, 1948) **42** *Microlestes discoidalis* (Fairmaire, 1892) **43** *Microlestes glabrellus* (Reitter, 1901) **44** *Microlestes infuscatus fragilis* Mateu, 1956 **45** *Pseudomesolestes Brittoni* (Mateu, 1956) **46** *Pseudomesolestes quadriguttatus* Mateu, 1979 **47** *Zolotarevskyella rhytidera* (Chaudoir, 1876).

##### Description.

Small beetles (Fig. 34), TBL 2.75–3.30 mm. *Color*: Frons and vertex black; clypeus, labrum, thorax, posterior half of epipleurae and dorsum and ventrum of head dark brown; elytra -dark brown with pale testaceous inverted comma shaped spot at humeri and oval shaped after half of elytra, not reaching the lateral and apical margins; legs, mouthparts, antennae, ventrum of abdomen and anterior half of epipleurae pale testaceous. *Microsculpture*: Head, clypeus, labrum, pronotum and elytra with irregular isodiametric pattern, neck with suppressed microlines; ventrum of head, thorax, and abdomen with microlines. *Head*: Almost as long as wide HL 0.58–0.71 mm and HW 0.59–0.67 mm, as wide as pronotum, tempora short (Fig. 20). *Pronotum*: Transverse, PW 0.56–0.64 mm PL 0.47–0.53 mm, narrowed posteriorly, sinuate before basal angles, base of pronotum straight in the middle with weak angles (Fig. 20). *Elytra*: Broadened posteriorly, EL 1.56–1.77 mm, EW 1.06–1.35 mm; intervals II with few scattered fine punctures, claws smooth. *Abdomen*: apical margin of last sternum in both males and females notched in the middle. *Aedeagus*: Shape of aedeagus (Fig. 46), AL 0.58 mm; in lateral view, aedeagus slightly curved dorsally, straight ventrally, broad from base to apical lamina; apical lamina narrowed, elongate and slightly curved before end with a small tooth dorsally; base of aedeagus also with a small tooth; internal sacs finger-like.

##### Affinities.

*Pseudomesolestes brittoni* is the only other species recorded from Saudi Arabia and is close to *P. quadriguttatus* in shape of head and pronotum, but can be distinguished by elytra of *P. quadriguttatus* considerably widened posteriorly with pale testaceous inverted spot at humeri and round spot after middle, legs completely pale testaceous, aedeagus with three elongate endophallus armatures.

##### Ecological notes.

This species was collected from hilly and mountainous zones of 892–2761 m elevation range (Fig. 53). It was collected during day time from root zones of superficial vegetation and small shrubs, while during night it fly to UV–light. The species was collected during January, February, March, September, and December.

##### Geographical distribution.

It is endemic to Saudi Arabia (Mateu 1990, Kabak 2017).

#### Zolotarevskyella

Taxon classificationAnimaliaColeopteraCarabidae

Mateu, 1953

##### Type species.

*Blechrus rhytiderus* Chaudoir, 1876.

This genus represents the subtribe Dromiusina by only three species (Anichtchenko 2017), two species *Z. afghan* Mateu, 1976 and *Z. rhytidera* (Chaudoir 1876) have been reported from Palaearctic region (Kabak 2003). This genus can be differentiated from other genera in the subtribe by combination of following characters: labrum transverse anteriorly; mentum without median tooth; last labial and maxillary palpomeres fusiform; antennomeres II as long as III, pubescence starts from the antennomeres III; eyes large, temples long; pronotum with longitudinal furrows in the middle; pronotum narrowed posteriorly and weakly sinuate; base of pronotum lobate, weakly incised towards hind angles; apex of elytra transversally truncates; tarsomeres I longer than V in hind legs. *Zolotarevskyella rhytidera* was described from Egypt (Chaudoir 1876) and then was reported from Saudi Arabia (Mateu 1986).

#### Zolotarevskyella rhytidera

Taxon classificationAnimaliaColeopteraCarabidae

(Chaudoir, 1876)

Figures 2 , 21 , 36 , 47 , 54

Blechrus rhytidera Chaudoir, 1876: 374.

##### Type locality.

Egypt, Upper Egypt.**Type depository**. Holotype in MNHN.

##### Material examined.

Total 32 specimens: Al Baha: 1♀, “KSA, Al Makhwa, Wadi Aleep, 20°10.695'N 40°68.556'E Alt. 455 m, 16.X.2014, (HP), I. Rasool”. 1♀, “Shada Al Aala, 19°50.710'N 41°18.267'E Alt. 1474 m, 18.X.2014, (PT), H. Al Dhafer, M.S. Abdel-Dayem, H. H. Fadl & I. Rasool”. 1♀, “19°50.391'N 41°18.634'E Alt. 1562 m, 3.XI.2013, (HP), I. Rasool”. Asir: 1♂, 1♀, “Saloos Al Manzar, Wadi Baqrah, 18°47.977'N 42°01.375'E Alt. 425 m, (HP), Al Dhafer H”. 1♂, “Al Magardah, Wadi Wabah, 19°14.911'N 41°47.255'E Alt. 402 m, 11.X.2013, (LT), I. Rasool, M. Al Harbi, S. Soonbati & S. Khan”. 1♀, “Al Hubail, Wadi Reem, 17°52.475'N 42°16.533'E Alt. 156 m, 20.X.2014, (HP)., 3♂, 4♀, “18°03.284'N 42°13.407'E Alt. 354 m, (HP)., 2♂, 1♀, “18°06.981'N 42°13.939'E Alt. 451 m, (HP), I. Rasool”. 1♂, 1♀ “18°06.981'N 42°13.939'E Alt. 451 m (LT), I. Rasool & M. Al Harbi”. Jazan: 1♂, “Adarab, Wadi Samar, 17°34.103'N 42°24.593'E Alt. 64 m, 24.II.2015, (HP)., 1♀ “Saybia, Saybia-Abu Areessh Road, 17°04.252'N 42°47.052'E Alt. -5 m, 24.II.2015, (HP), I. Rasool”. 1♀, “KSA, Fayfa, Al Abasia, 17°15.831'N 43°60.498'E Alt. 1770 m, 20.III.2014, (LT), S. A. El Sonbati”. 1♂, “Agricultural Research Station, 17°28.671'N 43°14.39'E Alt. 879 m, 6.IV.2013, (HP)., 1♀, “Wadi Jora, 17°22.856'N 43°06.169'E Alt. 419 m, (HP), M.R. Sharaf” [**KSMA].** 1♀, “19°50.391'N 41°18.634'E Alt. 1562 m, 3.XI.2013, (HP), I. Rasool”. 1♀, “18°03.284'N 42°13.407'E Alt. 354 m, (HP)” [**RMNH].**

##### Description.

Small parallel sized beetle, whole the body glossy (Fig. 36), TBL 2.38–2.90 mm. *Color*: Head and pronotum black; mouthparts, antennae, femora, anterior, lateral, apical margins of elytra and whole the ventrum of the bodydark brown; rest of elytra ferruginous; tibiae and tarsomeres pale testaceous. *Microsculpture*: Labrum, with irregular micro cells, clypeus and elytra with isodiametric mesh pattern, pronotum with isodiametric mesh pattern laterally; whole the ventrum of body with transverse microlines. *Head*: Almost as long as wide, HL 0.58–0.66 mm, HW 0.56–0.61 mm; dorsum of vertex and frons with longitudinal irregular longitudinal ridges; eyes moderate with short temples (Fig. 21). *Pronotum*: Slightly transverse, as wide as head, PW 0.57–0.62 mm, PL 0.46–0.50 mm, pronotum narrowed posteriorly; base of pronotum almost rounded with small basal angles; dorsum with longitudinal furrows at the middle (Fig. 21). *Elytra*: Parallel sized, EL 1.28–1.35 mm, EW 0.75–0.81 mm; apex transversally truncate, claws smooth. *Abdomen*: apical margin of last sternum bi–setose in both males and females, rounded in females slightly incised in males. *Aedeagus*: small, (Fig. 47) AL 0.72 mm; in lateral view it is curved dorsally and ventrally; narrowed at both ends, broad in the middle; ventral margin wavy; apical lamina in the middle, elongate apically; endophallus armature with a large hook.

##### Ecological notes.

It was recorded from various range elevation from 156–1770 m (Fig. 54). It is found among small vegetation and weeds and under the gravels near water streams associated with collembolans, spiders, Hemipteran, *Microlestes*, *Eremolestes*, *Tilius*, and *Apristus* species.

**Figure 48.**
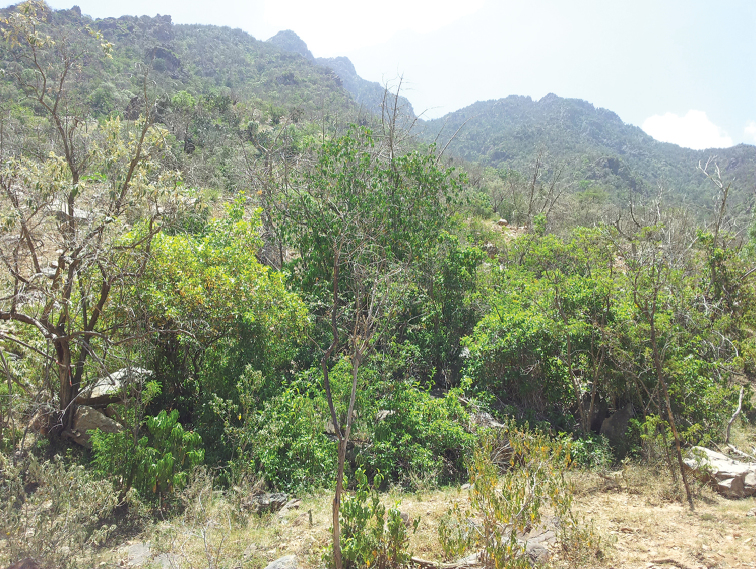
Photograph of the type locality for *Dromius saudiarabicus* sp. n. at Rayda Nature Reserve, Abha, Asir Province, southwestern Saudi Arabia at an elevation of 1897 m.

**Figures 49–54.**
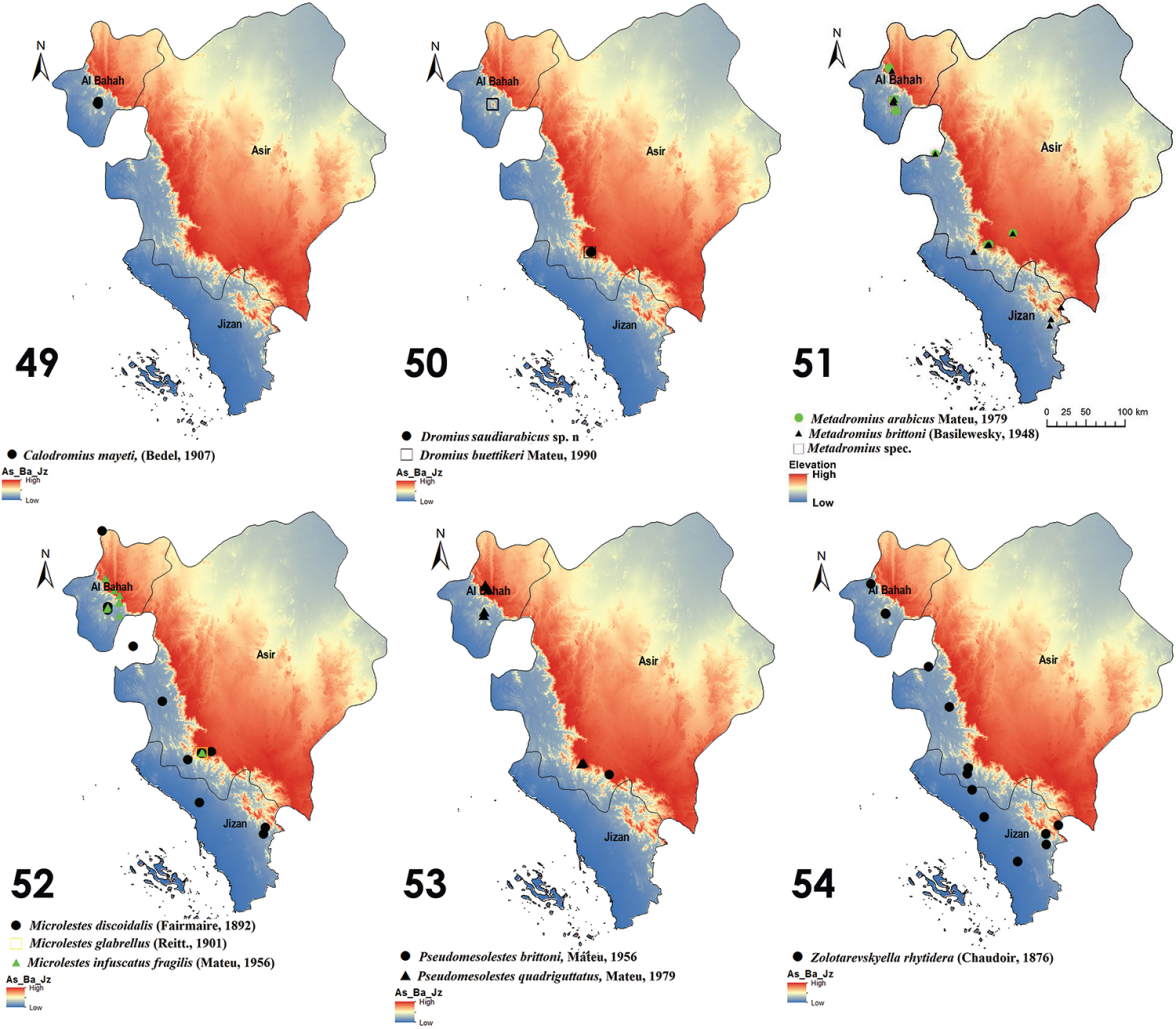
Distribution of Dromiusina species in Southwestern Saudi Arabia **49** *Calodromius mayeti* (Bedel, 1907) **50** *Dromius saudiarabicus* sp.n. and *D. buettikeri* Mateu, 1990 **51** *Metadromius arabicus*, Mateu, 1979, *Metadromius brittoni*, (Basilewesky, 1948) and *Metadromius* spec. **52** *Microlestes discoidalis*, (Fairmaire, 1892), *Microlestes glabrellus*, (Reitt. 1901) and *Microlestes infuscatus fragilis*, Mateu, 1956 **53** *Pseudomesolestes brittoni*, Mateu, 1956 and *Pseudomesolestes quadriguttatus*, Mateu, 1979 **54** *Zolotarevskyella rhytidera*, (Chaudoir, 1876).

##### Geographical distribution.

This species was described from Egypt (Chaudoir 1876) and also known from Saudi Arabia, Senegal and Yemen (Mateu 1986, Kabak 2003, 2017). This range exemplifies Saharo – Sahelo – Arabian chorotype.

## Supplementary Material

XML Treatment for Calodromius

XML Treatment for Calodromius mayeti

XML Treatment for Dromius

XML Treatment for Dromius saudiarabicus

XML Treatment for Dromius buettikeri

XML Treatment for Metadromius

XML Treatment for Metadromius arabicus

XML Treatment for Metadromius brittoni

XML Treatment for Metadromius

XML Treatment for Microlestes

XML Treatment for Microlestes discoidalis

XML Treatment for Microlestes glabrellus

XML Treatment for Microlestes infuscatus fragilis

XML Treatment for Pseudomesolestes

XML Treatment for Pseudomesolestes brittoni

XML Treatment for Pseudomesolestes quadriguttatus

XML Treatment for Zolotarevskyella

XML Treatment for Zolotarevskyella rhytidera
